# The content of meta‐supervision in a nursing educational context

**DOI:** 10.1002/nop2.220

**Published:** 2018-12-13

**Authors:** Marianne Kisthinios, Elisabeth Carlson

**Affiliations:** ^1^ Department of Care Science, Faculty of Health and Society Malmö University Malmö Sweden

**Keywords:** clinical supervision, content, meta‐supervision, nursing, theory

## Abstract

**Aim:**

The aim of this article was to illuminate the content of meta‐supervision of clinical supervisors active in a nursing programme delivering clinical supervision to nursing students in southern Sweden. The purpose of clinical supervision is to strengthen and develop the professional role through increased self‐awareness.

**Design:**

A qualitative, descriptive study was conducted analysing the documentation of 117 meta‐supervisory situations.

**Methods:**

Over 100 handwritten documented sessions, during 10 years of meta‐supervision, were analysed using content analysis.

**Results:**

The content of meta‐supervision consisted of three theoretical aspects: psychological aspects, pedagogical aspects and nursing aspects. To employ competent meta‐supervisors, the meta‐supervisor should have documented in‐depth knowledge of psychology, pedagogy and a good knowledge of the nursing context.

## 1 INTRODUCTION

Clinical supervision (CS) in nursing involves developing and strengthening the professional role of the nurse, in favour of providing the patient with safe care of high quality. CS is an educational model, which assumes that every person has the inherent ability to reflect on thoughts, feelings and actions based on personal experiences in pursuance of increased self‐awareness. The aim of CS is to strengthen and develop the professional role through increased self‐awareness. CS is based on the participants' narratives and theoretical perspectives such as nursing, ethics, group dynamics and leadership (The Swedish Society of Nursing & the Section for CS, 2015a). CS is frequently referred to in the literature but not well defined. Lyth (2000) proposed a definition of CS based on a concept analysis:

Clinical supervision is a support mechanism for practicing professionals within which they can share clinical, organizational, developmental and emotional experiences with another professional in a secure, confidential environment in order to enhance knowledge and skills. This process will lead to an increased awareness of other concepts including accountability and reflective practice. (p. 728)

Clinical supervision has three functions: the formative function, the restorative function and the normative function (Proctor, 2001). Brunero and Stein‐Parbury (2008) reported CS giving support and stress relief for nurses (restorative function) and also being a way to promote professional accountability (normative function). They also showed that CS promoted competence and knowledge development (formative function). The results of the study showed that all three functions, restorative, normative and formative were apparent. The restorative function was the most, although slightly, expressed (ibid.).

The content of CS was identified in a review by Pearce, Phillips, Dawson, and Leggat (2013). Three themes were identified; reflective practice, task‐oriented content and stress management. As for the content of reflective practice, it mostly concerned the meanings of behaviour, increased recognition and processing of the clinician's cognitions and emotional reactions in practice. The task‐oriented content referred to the activities that took place in CS sessions directed at specific objectives or had a task/solution focus. The final content of CS was that of stress management, this content had to do with sharing feelings of work‐related stress that in turn provided relief (ibid.). As for the theoretical perspectives in use, Berg and Kisthinios (2007) showed that the clinical supervisors in nursing often used and combined different theoretical perspectives with origins in nursing, pedagogy and psychology, although many clinical supervisors where insecure about the matter.

As clinical supervision has become a natural part of nursing in many organisations, the clinical supervisors, in turn, have had an increased need for a forum where they can discuss and reflect on experiences from clinical supervision. This forum and process is called meta‐supervision (Lund‐Jacobsen & Widsell, 2000). There is currently little research on meta‐supervision or supervision of supervisors. Teslo (2001) believes that meta‐supervision has many similarities with clinical supervision, but instead of focusing on nursing situations, the focus should be on the delivery of clinical supervision (ibid.). However, Elshaug Wik and Bruland Vråle (2007) have been active in the field. In an article on meta‐supervision they define the term:

Meta‐supervision is a systematic, professional and personal learning‐ and growth process that has its root in the supervisor's personal and professional development history. In meta‐supervision, a dialogue, based on acceptance, between the clinical supervisor and the meta‐supervisor may lay the foundation for a deeper understanding of clinical supervision. In the process, knowledge and experience are helping aids for a reflective recognition of one’s own clinical supervision and a deeper understanding of clinical supervision practice. (p. 41)

## 2 BACKGROUND

Arvidsson (2004) has touched the subject of meta‐supervision as she states that clinical supervisors need guidance on their clinical supervision, so‐called meta‐supervision to strengthen their own supervisory skills. She continues; in meta‐supervision, the method used is reflection, and focus is on the performed clinical supervision. Current topics may concern structure and frameworks, content, the relationship between theory and practice and the group dynamics (ibid.).

Meta‐supervision is also being discussed by Vråle (2000). They argue that the purpose of meta‐supervision is reflecting on the role of the clinical supervisor. Reichelt and Skjerve (2004, 2004) explore which areas or aspects that may be subject to reflection in meta‐supervision. These areas/aspects are; the commitment of the clinical supervisor, the relational aspects in clinical supervision, the meta‐theoretical perspective and the clinical supervisors awareness of his/her role as a clinical supervisor. Finally, they mention central aspects of the process of clinical supervision as being subject to reflection (ibid.).

The Swedish Board of Clinical Supervisors in Nursing states that taking on the mission as a meta‐supervisor is a matter of trust. Recruitment takes place through skills achieved in practice and the trust the meta‐supervisor may gain in being able to supervise supervisors. Therefore, regular postgraduate training is not specifically recommended. There is an ethical responsibility when taking on assignments as a meta‐supervisor. Thus, as a meta‐supervisor, should be able to clearly formulate goals for meta‐supervision, responsibilities, what quality the meta‐supervisor is aspiring to achieve, who and what values he/she represent. It is a matter of professionalism and personal qualities that makes a meta‐supervisor. Furthermore, good judgement, motives and awareness are essential in becoming a meta‐supervisor (The Swedish Society of Nursing & the Section for CS, 2015b).

Research into the content and the theoretical underpinnings of meta‐supervision in a nursing context is sparse. In fact, very little is known about not only the content, but also the theoretical perspectives, its function, outcomes, occurrence and process. A literature search in CINAHL, ERIC, Medline, PsycINFO and Sociological Abstracts made it evident that the scarcity was not only to be found in nursing but also in educational, social work, occupational therapy and the counselling domain of scientific publications. This article will provide the answer to the following question: What is the content and theoretical perspective in use in meta‐supervision as presented by 10 years documentation of meta‐supervision sessions with clinical supervisors in a nursing programme in the southern part of Sweden?

## 3 AIM

The aim of this article is to illuminate the content of meta‐supervision as documented by a meta‐supervisor during 10 years of meta‐supervision of clinical supervisors active in a nursing programme delivering CS to nursing students in southern Sweden.

## 4 METHODS

A qualitative, descriptive study was conducted analysing the documentation of 117 meta‐supervisory situations. As the first author (MK) is involved as the meta‐supervisor and the second (EC) is a clinical supervisor, we acknowledged the risk for bias possibly influencing the participants in an interview situation, thereby our decision was to use the documentation of the above‐mentioned situations. This procedure has previously been described as sufficient when exploring a phenomenon summarizing the participants’ experiences (Bengtsson & Carlson, 2015).

### 4.1 Settings

At a university in southern Sweden, all nursing students participate in CS two to four times a semester. About seven to nine students are supervised by the same clinical supervisor during the 3‐year undergraduate nursing programme. The participants in the groups remain the same although occasionally a student will drop out or a former student will drop in. The model in use since 1993 is a model designed by Lindell (2014). To support the clinical supervisors in turn, meta‐supervision has been offered twice a semester (1.5 hr/session). Time has been allocated by the employer for the participation of the supervisors. On these occasions, the supervisors meet in groups following the same model of clinical supervision as the students. In short, the model used in this meta‐supervision consists of 10 steps modified after and very close to Lindell's model (2014):

1. “Warm up”
2. All group members, in turn, describe situations they want/need to reflect on
3. The group members choose which situation to process
4. The “case holder” presents his/her situation based on his/her thoughts, feelings and actions
5. Questions are asked to the case holder to clarify the situation presented
6. The participants in turn reflect on the case presented by clarifying what they thought, felt and would have done in the same situation as originally presented
7. The case holder reflects on what he/she has learned and can perhaps do differently the next time when encountering the same situation
8. Open reflection to add or change any thoughts on the situation presented
9. When time allows; all group members reflect on what they have learned
10. Written documentation by meta‐supervisor

In meta‐supervision, the clinical supervisors are advised not to disclose the identity of the group members.

### 4.2 Data collection

As mentioned, the data consisted of written documentation derived from 117 meta‐supervision situations presented by the group participants in three clinical supervision groups during 2007–2017. All the written documentation was collected by one meta‐supervisor, in this case the first author (MK). Each group had approximately eight group members. The handwritten documentation collected by the meta‐supervisor during these years consisted of 39 pages with brief documentation such as the date, name of the participants for each and every session and the overall content of the session.

### 4.3 Analysis

To illuminate the themes processed in meta‐supervision, the content of the sessions were analysed by using manifest content analysis as described by Graneheim and Lundman (2004). The entire text was read, to get a feeling of the content. Phrases containing information relevant to the aim were picked out. Meaning units were cut out and condensed to shorten the text whilst still retaining the entire content. The condensed sentences were coded and grouped into categories reflecting the central content of the text. Finally, themes were formulated and presented as shown in the result. The first and last author (MK, EC) performed the analysis in parallel to ensure reliability. The chosen content that is presented in the analysis was discussed and agreed on to ensure credibility.

### 4.4 Ethics

According to the Codex (2018) set by the Swedish Research Council ethical approval was not needed for this study as the content of the written documentation did not cover issues related to sexual‐, political‐ or religious orientation. Nevertheless, confidentiality is pivotal to CS and meta‐supervision, therefore the ethical aspects of this qualitative study was very important. Written information on the study objective and the method was given by e‐mail to each clinical supervisor that had participated in meta‐supervision. The clinical supervisors were ensured of confidentiality and that data were to be presented on group level. Because of the delicate matter discussed in the groups and to approve and validate the results, the clinical supervisors were presented with preliminary results. They were asked to give their feedback and thereby secure that nothing of sensitive content would be revealed. The clinical supervisors made some comments in relation to an identified risk of disclosing the identity of students, and the results were corrected accordingly. After being corrected, the results were again presented to the clinical supervisors, following this procedure none of the clinical supervisors opposed to the results or withdrew their approval.

### 4.5 Definitions

*Meta‐supervisor*; the person supervising clinical supervisors in meta‐supervision. For this study, the meta‐supervisor was a nurse lecturer and an experienced clinical supervisor.

*Clinical supervisor*; the clinical supervisor is either a clinical nurse or a lecturer, supervising nursing students in clinical supervision in a nursing programme.

*Group member*; nursing students participating in compulsory clinical supervision during their nursing education.

## 5 RESULTS

### 5.1 Psychological aspects (69 situations)

The uttermost common content in meta‐supervision was psychological.

#### 5.1.1 “Challenging” group members (29 situations)

The most common topic the clinical supervisors reflected on in this category was the group members taking up too much space in the group or being very quiet. Another topic that the clinical supervisors worried about and raised in meta‐supervision was the group members perhaps unsuitable for the profession. Another common theme raised in meta‐supervision was the defying group members, that is, those who defied the supervisor in their leadership. Another shared subject for reflection concerned group members who perhaps had some psychological challenges and in what way the clinical supervisors should handle it. Some group members were perceived as too unformed for the nursing profession by the clinical supervisors, and this in turn led to the need for the clinical supervisors to reflect on the consequences. Another topic that the clinical supervisors could find difficult to handle was the reluctant group member who for various reasons did not want to be supervised or join the group.

#### 5.1.2 Group psychological aspects (18 situations)

Other aspects in meta‐supervision were of group psychological nature. The most common themes were the issues surrounding the silent groups and the impact of new members on the group process. Other topics brought up in meta‐supervision were groups that did not develop as one could expect or which did not show an interest in clinical supervision. A theme that could also be shared in meta‐supervision was the negative impact of group members’ absence on the group process. There were also positive aspects raised by the clinical supervisors such as the impact a new reflective, positive group member had on the group, as the new group member affected a whole group to become more open and willing to reflect. Another positive aspect the clinical supervisors talked about was the characteristics of a group that was completely self‐driven and the constructive outcomes related to that. On some occasions, the clinical supervisors reflected on how they should act when the group members wanted them to be the experts. Finally, the clinical supervisors also reflected on the challenges to the group process when group members were perceived as being different in the group due to their difficulties in expressing themselves or due to a difference in age compared with the other group members.

#### 5.1.3 Emotional aspects of the clinical supervisors (15 situations)

A common theme in meta‐supervision concerned the emotions of the clinical supervisors. Whilst supervising, the clinical supervisors often had to take into consideration their own feelings and emotions. The most common feeling was the feeling of being afraid or worried. The most common worry concerned the so‐called “case‐draught,” that is the group members not having any cases to reflect on making clinical supervision impossible and leaving the clinical supervisor without substance for the clinical supervision session. Sometimes, the clinical supervisors could feel separation anxiety and processed that, as they found it difficult to let the group go. Another common theme reflected upon in meta‐supervision, was the weariness of the clinical supervisor. A feeling originating from many years of supervising and experiencing some groups as being unable to mature or develop. In turn, that made the clinical supervisor feeling unable to carry on. This feeling of not being able made the clinical supervisors feel sad as they considered clinical supervision a very important part of nursing education. Other sessions concerned feeling irritated because of some group members in the group. Finally, clinical supervisors could feel omitted during supervision sessions whilst feeling exposed due to having shared something important and not having the response anticipated by the group members in the group.

#### 5.1.4 Emotional aspects of the group members in CS (7 situations)

Another theme raised in meta‐supervision was the emotions of the group members in the CS groups (i.e., nursing students). Two themes were evident, the first aspect concerned private problems that spread from the private lives of the group members to the sessions in CS. The other concerned the group members' suffering from seeing their patients suffer. In particular, the most painful situations presented were about the suffering of children and the suffering of suicidal patients. The clinical supervisors also at times reflected on cases where group members had been shaken by experiencing difficult patient situations.

### 5.2 Pedagogical/methodological aspects (37 situations)

The pedagogical or methodological aspects of CS were also a topic that the clinical supervisors reflected on. A recurring theme was the difficulties and possibilities that the model (Lindell, 2014) in use provided. Many sessions in meta‐supervision were used to reflect on the supervisory role of the clinical supervisors. Such as, to what extent the clinical supervisors should be participating in the reflections and to what extent the clinical supervisors should be "educators." The content of meta‐supervision was also largely assigned to teaching activities. It was a matter of pure training of the clinical supervisors. Teaching activities such as how to use “Structured fantasy” (Tveiten, 2013) “Reflecting teams” (Andersen, 2011), “Using images” (Tveiten, 2013) “Empathy training” (Englander, 2014; Englander & Robinson, 2009) and the use of “The pedagogic sun” (Tveiten, 2013) were common. At one session, a clinical supervisor shared an experience of using images in CS and the negative outcome of it. The clinical supervisors also provided each other with advice on how to optimize CS. Another reoccurring topic in meta‐supervision was how to distinguish the difference between the group members being private rather than personal. Some meta‐supervision sessions were devoted to discussing concrete solutions to the CS grading aspects, as CS is mandatory for the nursing students.

### 5.3 Nursing aspects (11 situations)

Nursing aspects were the least frequent content shared in meta‐supervision. Although on several occasions, the clinical supervisors raised the problem of adverse events that the group members reported in CS. In some cases, the clinical supervisors were unsure how to handle the situation and how to support the group members in reporting. For some clinical supervisors, it was difficult to know how to handle the issue of adverse events in nursing that was brought to CS and how not to feel powerless in these situations. At last, at some occasions the clinical supervisors raised the question of what to do when discovering that group members in the clinical supervision groups exposed themselves exercising poor nursing.

## 6 DISCUSSION

The result indicates that the content of meta‐supervision can be divided into two aspects. One aspect concerns the framework of CS (the pedagogical aspects) and the other aspect concerns the psychological processes and expressions (the psychological aspects) in turn, these aspects are based on a nursing context.

The results firstly showed that the content mostly related to psychological aspects arising from situations that the clinical supervisors experienced in their CS and secondly on pedagogical aspects. Nursing was included in the content the clinical supervisors lifted in meta‐supervision but did not play a prominent role. All types of supervision are an educational process with both an instrumental and an emotional aspect to consider (Pertoft & Larsen, 1991). Cognitive, affective and psychomotor domains are always involved in the learning process (Bloom, Masia, & Krathwohl, 1964) as learning is a collaborative process enhanced by social interactions between people. The social and psychological processes are equally important and mutually influential (Vygotsky, 1978).

The result is in line with Reichelt and Skjerve (2004, 2004) and Arvidssons (2004) thoughts on the content of meta‐supervision, except for the result indicating that the theoretical connections to practice were not highlighted in the meta‐supervision sessions. This also appears to be the case when Kisthinios (2017) evaluated this aspect in a report on meta‐supervision. In this report, it became evident that meta‐supervision provides clinical supervisors with support and engagement in the clinical supervisor role. It helped the clinical supervisors with the relational perspectives in CS and it made the clinical supervisors aware of their role in CS. Furthermore, it supported the clinical supervisors with the central/pedagogic aspects of the process. The clinical supervisors did not report any support in the opportunity to highlight theoretical perspectives related to the content of meta‐supervision (ibid.).

Lindell's model (2014) does not automatically give space for reflection and critical thinking when it comes to the theoretical assumptions underpinning the psychological or pedagogical aspects of meta‐supervision. One way to highlight the theoretical aspects could be giving explicit space in the model, given there is a need for the theoretical assumptions to be reflected on.

Unfortunately, it is difficult to find instructions on how meta‐supervision is conducted (Folmer, 1999); therefore, it is in general hard to know if this dimension is reflected on in meta‐supervision when using other models for meta‐supervision apart from the one used in this study.

All forms of supervision are based on interpersonal meetings. In meta‐supervision, it becomes clear that the content concerns how the clinical supervisors should handle themselves and the group members in CS, on a psychological level. The quality and content of meta‐supervision relies on the relational, emotional and cognitive aspects of the group being supervised, and it cannot be excluded that parallel processes occur between the meta‐supervisor and the clinical supervisors, which in turn may affect the content.

As mentioned, The Swedish Board of Clinical Supervisors in Nursing state that taking on the mission as a meta‐supervisor is a matter of trust and that recruitment takes place through skills achieved in practice. Therefore, The Swedish Board of Clinical Supervisors in Nursing do not specifically recommend regular postgraduate training. The result of this study points to another direction. In addition, deepened knowledge in psychological and pedagogical theoretical and practical perspectives seem to be needed. Apart from that, aspects of interest that might be included in the training of meta‐supervisors mentioned by Reichelt and Skjerve (2004, 2004) and discussed by the authors of the current study are as follows:

### 6.1 In‐depth understanding of critical thinking

Reflection and critical thinking is of crucial importance to all supervision. It is also one of its main objectives. Reflection might lead to critical thinking, which in turn may start processes that are controversial for example in an organization. Therefore, it is important for the meta‐supervisors to have an in‐depth understanding of not only its definition but also its consequences. Andrade Dias, Scherlowski Leal David, and Muniz da Costa Vargens (2016) suggest that through critical thinking “The speech will be more critical, more liberating, more explanatory, all reasons why thinking critically should be a practice encouraged...”.

### 6.2 Structuring and completion of the clinical supervisors own developmental work

As meta‐supervisors in general are experienced clinical supervisors and most possibly have been supervising for many years and under different circumstances, it is likely that they have identified areas and complex situations in clinical supervision that they need to explore and develop. During their training, it would be beneficial both for the clinical supervisor and for clinical supervision in general and meta‐supervision particularly to have the opportunity for in‐depth exploration of these areas of interest.

### 6.3 Meta‐communication

Meta‐communication on relations, actions and theory in CS is necessary for the meta‐supervisors. Ruesch and Bateson (1951) defined the term meta‐communication as “communication about communication.” The importance of meta‐communication goes far beyond the apparent fact that communication can be a topic of discussion; therefore, it is necessary to communicate about communication. Meta‐communication is a "new order" for communication and it can clarify some clearly complex, creative and deeply puzzling qualities of social interaction (ibid.). Meta‐communication provides unlimited possibilities to develop meta‐supervisors and/or meta‐supervision and contributes to opportunities to examine one's own practice.

### 6.4 Integrating theory and practice

In addition, understanding the possibilities and limitations of integrating theory and practice in meta‐supervision and CS is fundamental. On the one hand, it has to be discussed if it is meaningful to pursue the integration of theory and practice in the actual “real‐life” meta‐supervision or in clinical supervision in general, as it might interfere with the process of reflection during clinical supervision sessions. It might create tension for the group members having to declare on what theoretical ground they are making their statements. On the other hand, Watson, Burrows, and Player (2002) suggest that to be able to successfully implement theory in practice one must be able to reflect critically on one's own practice and the consequences of one's own actions.

### 6.5 The use of technology in distance CS and meta‐supervision

In the future, one way to carry out meta‐supervision may be by video‐conferencing since technology has advanced, and many clinical supervisors need to put substantial distances to attain meta‐supervision. Marrow, Hollyoake, Hamer, and Kenrick (2002) showed that clinical supervision could be conducted through video‐conferencing although there were some technical, accessibility problems and communication problems. Since this was in 2002, there is good hope for these problems having decreased. It is very possible that meta‐supervision also can benefit from this way of using technology.

## 7 LIMITATIONS

Limitations of the study should be discussed. Lincoln and Guba (1985) state that trustworthiness of a research study is vital to assessing its worth. The trustworthiness constitutes of four dimensions; credibility, transferability, dependability and confirmability. In this study, one might argue that the first author also was the one collecting the data and therefore may have had an impact on the credibility and the conformability. On the other hand, the second author was well in accordance with the interpretation of the results and did not participate in the documentation and interpretation of the meta‐supervisory sessions. As for the transferability, it is very possible that the findings are applicable in other contexts. Unfortunately, this study did not distinguish between the theoretical perspectives in use to understand the content only; but mixed the theoretical perspectives in use both in the content of CS and in the process of CS. Dependability is showing the consistency of the findings and the opportunity of them being repeated. Since this is the first published study of its kind, it would be valuable to conduct further research in a clinical setting with registered nurses who have participated in meta‐supervision.

Furthermore, the results only show the content of meta‐supervision as perceived by one meta‐supervisor supervising clinical supervisors in a nursing educational context. It is not certain that the results would be the same if this study were to be conducted with clinical supervisors supervising in a nursing context or in a different cultural context. It is also important to examine the role of the first author (MK) since she was also the meta‐supervisor collecting the data. There is some potential bias since it is this researchers perceived content of each clinical supervision session. One has to ask oneself how these sessions would have been understood and interpreted by another meta‐supervisor. It is also very possible that the content would be altogether different with a different meta‐supervisor since the meta‐supervisor has a potent role and might in various ways influence the content of meta‐supervision.

To employ competent meta‐supervisors, the meta‐supervisor should have documented in‐depth knowledge of psychology, pedagogy and a good knowledge of the nursing context. Further studies need to explore whether the content of meta‐supervision is the same in clinical supervision of students as of professional nurses.

## 8 CONCLUSION

In conclusion and to employ competent meta‐supervisors, the meta‐supervisor should have documented in‐depth knowledge of psychology, pedagogy and a good knowledge of the nursing context. Furthermore, it is suggested that the training of meta‐supervisors contains in‐depth understanding of critical thinking and a theoretical deepening and exploration of identified problematic areas and complex situations. Meta‐communicational skills should also be addressed with focus on relations, actions and theory in CS. In addition, understanding the possibilities and limitations of integrating theory and practice in meta‐supervision and CS is fundamental.

## CONFLICT OF INTEREST

None.
